# Effects of laparoscopy, laparotomy, and respiratory phase on liver volume in a live porcine model for liver resection

**DOI:** 10.1007/s00464-020-08220-0

**Published:** 2021-01-04

**Authors:** Hannes G. Kenngott, Felix Nickel, Anas A. Preukschas, Martin Wagner, Shivalik Bihani, Emre Özmen, Philipp A. Wise, Nadine Bellemann, Christof M. Sommer, Tobias Norajitra, Bastian Graser, Christian Stock, Marco Nolden, Araineb Mehrabi, Beat P. Müller-Stich

**Affiliations:** 1 Department of General, Visceral and Transplantation Surgery, Heidelberg University, Im Neuenheimer Feld 110, 69120 Heidelberg, Germany; 2 Department of Diagnostic and Interventional Radiology, Heidelberg University, Im Neuenheimer Feld 110, 69120 Heidelberg, Germany; 3 Division of Medical and Biological Informatics, German Cancer Research Center, Im Neuenheimer Feld 280, 69120 Heidelberg, Germany; 4 Institute of Medical Biometry and Informatics, Heidelberg University, Im Neuenheimer Feld 130.3, 69120 Heidelberg, Germany

**Keywords:** Operation planning, Soft tissue surgery, Respiratory phase, Liver volume, Computer assistance, Navigation

## Abstract

**Background:**

Hepatectomy, living donor liver transplantations and other major hepatic interventions rely on precise calculation of the total, remnant and graft liver volume. However, liver volume might differ between the pre- and intraoperative situation. To model liver volume changes and develop and validate such pre- and intraoperative assistance systems, exact information about the influence of lung ventilation and intraoperative surgical state on liver volume is essential.

**Methods:**

This study assessed the effects of respiratory phase, pneumoperitoneum for laparoscopy, and laparotomy on liver volume in a live porcine model. Nine CT scans were conducted per pig (*N* = 10), each for all possible combinations of the three operative (native, pneumoperitoneum and laparotomy) and respiratory states (expiration, middle inspiration and deep inspiration). Manual segmentations of the liver were generated and converted to a mesh model, and the corresponding liver volumes were calculated.

**Results:**

With pneumoperitoneum the liver volume decreased on average by 13.2% (112.7 ml ± 63.8 ml, *p *< 0.0001) and after laparotomy by 7.3% (62.0 ml ± 65.7 ml, *p *= 0.0001) compared to native state. From expiration to middle inspiration the liver volume increased on average by 4.1% (31.1 ml ± 55.8 ml, *p *= 0.166) and from expiration to deep inspiration by 7.2% (54.7 ml ± 51.8 ml, *p *= 0.007).

**Conclusions:**

Considerable changes in liver volume change were caused by pneumoperitoneum, laparotomy and respiration. These findings provide knowledge for the refinement of available preoperative simulation and operation planning and help to adjust preoperative imaging parameters to best suit the intraoperative situation.

Safe performance of hepatectomy, living donor liver transplantations (LDLT) and other major hepatic interventions rely on preoperative calculation and estimation of total and residual liver and graft volume. Liver resections are required to respect the anatomical segmentation of the liver, safety margins in oncological surgery, and resection lines guaranteeing that the residual liver volume and function is sufficient and oncological outcomes are adequate [1, 2]. Preoperative calculation and simulation of liver volume can assist the surgeon in identifying safe operation strategies with respect to the individual vascular, segmental and oncological characteristics [3]. Furthermore, intraoperative navigation may facilitate three-dimensional orientation of the surgeon and promises to help in the identification of at-risk structures and safe resection lines [4]. Navigation may be of particular benefit in minimally invasive surgery [5] where it may help compensate for the additional difficulties of the confined space of pneumoperitoneum, two-dimensional representation of the operating field and the unintuitive instrument handling. Intraoperative navigation requires a precise alignment of the preoperative imaging data to the intraoperative situation [5, 6]. In abdominal surgery, soft tissue movement and deformation causes the organs to change considerably in form, position and size [7]. These changes are the result of positioning [8], respiration [9], pneumoperitoneum [10], tissue dissection and iatrogenic manipulation [11, 12]. To compensate for this phenomenon there are two approaches: Intraoperative imaging and other methods of tissue localization [5, 6, 11, 13, 14], or biomechanical modeling, which tries to predict the tissue deformation based on calculations of the factors responsible for the deformation.

Here we report on a study with the following aims: 1. to analyze and quantify the influence of laparotomy and pneumoperitoneum for laparoscopy on liver volume; 2. to assess the effect of different inspiratory volumes on liver volume in the different operative states.

## Materials and methods

### Subjects

A total of ten pigs (German landrace, 20–34 kg) were analyzed. The study protocol was approved by the local Ethics Committee in Heidelberg, Germany and by the regional committee in Karlsruhe, Germany. The care and veterinary handling were carried out by the staff of the Interfaculty Biomedical Research Facility at Heidelberg University and complied with the recommendations outlined in the “Guide for the Care and Use of Laboratory Animals” prepared by the National Academy of Sciences and published by the National Institutes of Health [15].

The animals were fasted 12 h before the intervention. A modified anesthesia protocol was used based on Clutton et al. [16]. After premedication with azaperone (0.1 mg/kg), midazolam (0.1 mg/kg) and ketamine (15 mg/kg), the induction of anesthesia was carried out by intravenous midazolam (0.1 mg/kg) and ketamine (20 mg/kg). Anesthesia was maintained with intravenous midazolam (0.05 mg/kg) and ketamine (10 mg/kg). Pancuronium was used as needed. The animals were machine ventilated (*f* = 12/min, ventilation volume = 250–320 ml). The animals were positioned in a 0° supine position on a vacuum mattress which was firmly attached to a stretcher. This stretcher and vacuum mattress combination guaranteed full immobilization of the animal between scans [17]. The animals on the stretcher were left in place on a fixed position on the CT scan table for the entire duration of the experiments to rule out repositioning errors. The animals were under general anesthesia with machine ventilation during the entire experiments. At the end of the study the animals were euthanized using potassium chloride (150 mg/kg) as per protocol.

### Study design

Each pig was examined by Computed Tomography (CT) in nine different states. The CT scans were obtained for three respiratory states (full expiration, middle inspiration and deep inspiration) in each of three operative states (native, pneumoperitoneum and laparotomy). The CT scans were taken with a slice thickness of 2 mm and a 1 mm overlay with the SOMATOM Sensation™ 64 Row Dual Energy CT device (Siemens Corp. Erlangen, Germany). Before imaging 50 ml of the contrast agent Imeron®300 (Bracco Imaging Deutschland GmbH, Konstanz, Germany) was intravenously administered. CT scans were acquired 120 s after application of the contrast agent, which corresponded to the venous phase. For each respiratory state manually controlled breath-hold positions were realized. End-expiratory hold maneuver was performed to maintain full expiration. Our definitions for high tidal volume was 14 ml/kg (deep inspiration, approximately 400 ml) and for middle tidal volume was 7 ml/kg (normal inspiration, approximately 200 ml) based on previous studies [18–20]. Both inspiratory levels were maintained by occluding the respiratory port once the tidal volume was reached.

The pneumoperitoneum for laparoscopy was created using a Veress-needle (14-gauge) in the left lower quadrant of the abdomen. A standard pressure-controlled insufflation device was used to maintain an intra-abdominal pressure of 15 mmHg with CO_2_-insufflation. After imaging was completed with pneumoperitoneum the Veress-needle was removed and a standard midline laparotomy of 20 cm length was performed using a disposable scalpel. The data from the CT scans were transferred onto a mobile hard disk for the following evaluation. All pictures complied with the Digital Imaging and Communications in Medicine (DICOM) standard.

### Image processing and segmentation

**Fig. 1:**
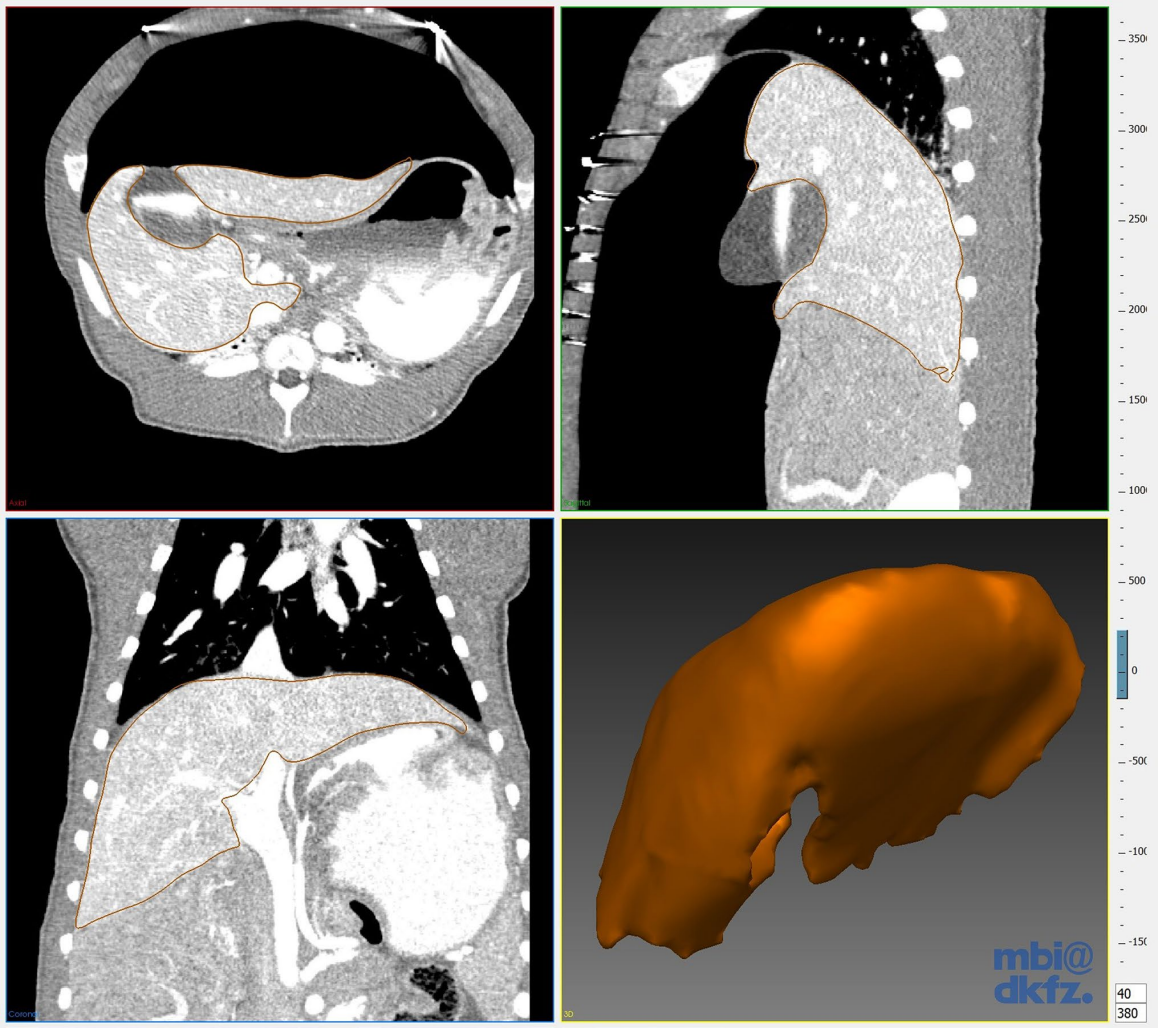
Manual segmentation of a computed tomography scan of the porcine liver with pneumoperitoneum in the Medical Imaging Interaction Toolkit (MITK). Axial view (top left), Sagittal view (top right), Coronal view (bottom left) and three-dimensional model based on the segmentations (bottom right)

The imaging data were post-processed using the Medical Imaging Interaction Toolkit (MITK) which was developed by the Division of Medical and Biological Informatics at the German Cancer Research Center in Heidelberg [21]. The segmentation process consisted of manually circumscribing the liver tissue in the transversal view for one slice in every eight slices. The slices between the manually segmented slices were interpolated by the MITK software (Fig. 1). Detailed information on the interpolation algorithm can be found in the MITK documentation [22]. The interpolated slices were checked manually and additional slices were manually segmented if the interpolation was not accurate. The vena cava, the extrahepatic portal vein and the gall bladder were consistently excluded from the segmentations. The end result was checked for correctness in each slice and in all three views (transversal, sagittal and coronal). Image segmentation was performed and cross-checked by two independent and specially trained professionals. Three-dimensional mesh models were generated from the segmentations of the liver using the MITK software. For each mesh model the corresponding volume was calculated using the open-source software MeshLab which was developed by the Italian Institute of Information Science and Technology and the Italian National Research Council. We used MeshLabs “Compute Geometric Measures” function [23].

### Statistical analysis

The data were evaluated using a hierarchical linear regression [24]. We examined the influence of the operative state (native, laparotomy, pneumoperitoneum) and the respiratory state (continuous predicator with levels expiration, middle inspiration and full inspiration) on the measured liver volumes. We analyzed liver volume changes depending on the operative state based on the interaction between operative state and respiratory state. Liver volumes of each animal were considered with a random factor. The data were indicated as mean ± standard deviation, if not otherwise specified. Significance level was set to *α* = 5% two-sided, without correction for multiple testing. Graphs were created using the plotrix package for R (R Foundation for Statistical Computing, Vienna, Austria).

## Results

### Total liver volume

The mean liver volume over all measurements was 792.9 ± 96.1 ml (minimum: 644.3 ml; maximum: 1034.6 ml).

### Operative state

**Fig. 2:**
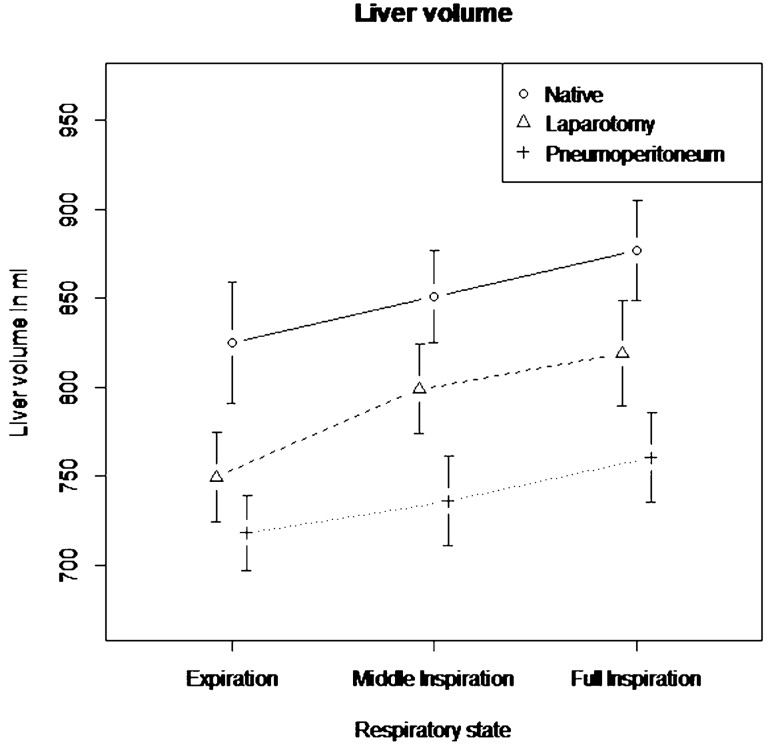
Liver volume stratified by operative and respiratory state in milliliters (ml) (mean ± standard error)

The mean volume in the native state was 851.0 ml ± 92.8 ml (minimum: 666.0 ml; maximum: 1034.6 ml), with pneumoperitoneum it was 738.4 ml ± 75.1 ml (minimum: 651.7 ml; maximum: 895.4 ml) and after laparotomy it was 789.1 ml ± 86.5 ml (minimum: 644.3 ml and Maximum: 1002.3 ml). With pneumoperitoneum the liver volume decreased by 13.2% or 112.7 ml ± 63.9 ml (95%-CI 88.9–136.6 ml, *p *< 0.0001) compared to the native state. After midline laparotomy the liver volume was still reduced by 7.3%, or 62.0 ml ± 65.7 ml (95%-CI 37.4–86.5 ml, *p *= 0.0001) compared to the native state but was not significantly higher than with pneumoperitoneum (*p* = 0.18 (Fig. 2).

### Respiratory state

The mean volume with full expiration was 764.3 ml ± 95.1 ml (minimum: 644.3 ml; maximum: 1034.6 ml), with middle inspiration it was 795.4 ml ± 90.9 ml (minimum: 663.9 ml and Maximum: 971.5 ml) and with full inspiration it was 818.9 ml ± 97.2 ml (minimum: 659.7 ml and Maximum: 1019.2 ml). Inspiratory volumes had a significant influence on liver volume. With middle inspiration liver volume increased by 4.1% or 31.1 ml ± 55.8 ml (95%-CI 10.3–51.9 ml, *p* = 0.166) and with full inspiration by 7.2% or 54.7 ml ± 51.8 ml compared to expiration (95% CI 35.4–74.1 ml, *p *= 0.007) (Fig. 2).

## Discussion

Laparotomy, Pneumoperitoneum and respiratory states had a significant influence on liver volume. Compared to the native state the liver volume decreased most with pneumoperitoneum (13.2%) and less after midline laparotomy (7.3%). With full inspiration liver volume increased more (7.2%) than with middle inspiration (4.1%) compared to expiration. The highest change in liver volume was produced by pneumoperitoneum in the present study. Earlier studies showed that liver volume may vary up to 33% between perfused and non-perfused livers [25–27]. In the literature several cardiovascular changes are reported with pneumoperitoneum: decreased cardiac output and stroke volume, decreased venous return, increase of systemic resistance and adverse splanchnic circulatory effects [28, 29]. Concerning the liver, decreased hepatic perfusion has been reported with pneumoperitoneum [30–32]. Decreased portal [33–35] and hepatic [36–39] blood flow was reported in several studies, although some studies did not find significant change of portal flow [30, 40]. The diameter of the portal vein and mean luminal area of the portal vein were also found decreased in other studies, which is consistent with a decrease of the portal vein flow with pneumoperitoneum [41, 42]. The pressure of the pneumoperitoneum was also shown to decrease the diameter of the Inferior vena cava [43–46] and to cause a decrease of the blood flow in the vena cava [42]. These factors are in line with a decreased blood volume in the liver during pneumoperitoneum which likely caused the decrease liver volume. One possible explanation is that the pressure created in the abdominal cavity lead to a decrease in hepatic perfusion which finally caused the consistent decrease in liver volume. A study by Moyano-Cuevas et al. [41] described an increase of liver volume after insufflating the abdomen with 14 mmHg of CO_2_. This contradicts our findings; however, our results seem to be more in line with the cardiovascular and other abdominal changes occurring during pneumoperitoneum [46].

The reduction in liver size from native volume to laparoscopy is most likely explained from the increase in intra-abdominal pressure during laparoscopy. However, the reduction in volume between native state and laparotomy was an unexpected result. To better assess the change in intra-abdominal pressure differences between the three modalities of native, laparoscopic and open, a follow-up experiment was conducted whereby the intra-abdominal pressure was measured via bladder catheter, at five measurements per operative state per subject (*n* per state = 25). The results are summarized in Table 1 and show a significant difference in intra-abdominal pressure between both native state and pneumoperitoneum, and laparotomy and pneumoperitoneum. The change in liver volume in laparotomy could also be an effect of the physiological surgical stress response, as sympathetic activation could lead to decreased perfusion of the liver [47–49]. A further possible reason for a smaller volume during laparotomy is due to the sequence in which the measurements were conducted. As median laparotomy was performed after pneumoperitoneum, the liver volume had already been reduced through the increased intra-abdominal pressure. Whether this was the causative factor, however, was not assessed because no further CT scans were performed after prolonged laparotomy to check for a possible later increase in liver volume. This is certainly a limitation of the current study, and should be taken into consideration when designing future experiments attempting to evaluate differences in surgical modalities. It nevertheless does not detract from the main argument of the current study that liver volume is decreased by increased intra-abdominal pressure, and is a further indication that the pneumoperitoneum decreases perfusion, which would require more time to return to previous volume than simple tissue displacement or compression.

**Table 1:** Comparison of intravesicular pressure of the measured operative states

Intravesicular pressure (*n* = 75)	Native	Pneumoperitoneum	Laparotomy
Mean pressure [mmHg]	5.95	11.73	7.08
Standard deviation [mmHg]	1.68	1.80	2.68

Comparison	*p* value
Native vs pneumoperitoneum	**< 0.001**
Native vs Laparotomy	0.059
Pneumoperitoneum vs laparotomy	**< 0.001**

Significant *p*-values in bold

Liver volume change after laparotomy has been studied for discrepancies between preoperatively calculated and intraoperatively measured liver graft volumes in liver transplantation. Lemke et al. found a conversion factor for calculated graft weight and actual graft weight (0.75 of virtual measurement) of the non-perfused graft which improved measurement accuracy [50]. Karlo et al. found a conversion factor for CT (0.85 of virtual measurement) and MRI (0.78 of virtual measurement) to adjust preoperatively calculated resection volumes to actual intraoperative resection volume [51]. This compares similar to our findings of 13.2% (pneumoperitoneum vs native expiration) and 7.3% (laparotomy vs native expiration) changes in liver volume. The volume decrease in the present study was expectedly less than in the aforementioned studies by Karlo and Lemke since the present study was completed in situ in perfused organs versus the resected non-perfused organs in above mentioned studies. These findings should be discussed regarding clinical relevance. Primarily, liver volume as measured preoperatively is the metric used when deciding safe resection volumes in partial hepatectomies, as well as determining adequate volumes for transplantation surgery. Guidelines exist with cut-offs and recommendations for safe resection/transplantation, but these are also dependent on surgeon experience and individual consideration. Changes in liver volume between native state, laparoscopy, and laparotomy will in the future be important with the introduction of intraoperative imaging and assistance systems that aim for high resolution estimates of postoperative outcomes, e.g., of future remnant liver volume. This accuracy will then also depend on the intraoperative change of liver volume depending on the mode of surgery. A change in liver volume of up to 13% in the current study would be important to consider when critical decisions have to be made regarding resectability or alternative treatment strategies. The clinical application hence lies in the influence on intraoperative decision-making and the influence on surgical outcome, such as overestimating liver remnant volume in resection or misjudging functional size of live donor liver segments in preoperative planning. On the other hand, the use of navigation systems with intraoperative tracking and segmentation of liver volume and position according to preoperative images would also be influenced by changes in liver volume, form, and position. In regards to preoperative planning, differences in preoperative measured volume and in situ volume have been recorded previously. In volumetric analysis of living donor transplant livers, Baskiran et al. described significant reductions between estimated and intraoperative volumes [52], and correctly stressed the need for surgical awareness to prevent small-for-size and large-for-size errors. The current study should underline this finding, and further emphasize that liver volume must be critically re-evaluated in the intraoperative setting, especially in laparoscopic procedures.

Liver volume increased with inspiration in the present study. A possible explanation of the increase in volume could be the decrease of central venous return and thus a decrease of venous return from the liver blood pool due to the positive pressure ventilation mandatory with general anesthesia in this experimental model [53]. However, other studies showed no significant effect of mechanical ventilation and positive end-expiratory pressure on liver blood flow [54–56]. The reason for this discrepancy therefore warrants further investigation.

A further clinical aspect to be discussed would be a critical evaluation of preoperative liver CT scans in regards to resection planning. Currently, CTs are performed at maximal inspiration. This may result in the liver measurements being significantly larger than in situ intraoperatively, and could result in overestimation of either required resection area or of the volume of the remaining liver. A possible clinical recommendation could be to perform the CT at maximal expiration for better preoperative assessment of liver volume; however, the small sample size and experimental nature of the current study limit a direct clinical translation. Further evaluation in a clinical setting would be warranted when intraoperative imaging modalities with volumetric assessment become more widely available, such as intraoperative CT or MRI imaging. The current study however shows that such studies should be performed in humans to avoid a mismatch between preoperative planning and intraoperative situations in such future scenarios.

Additionally, a significant change in volume and liver perfusion may also result in a significant shifting of structures in the liver, such as the exact location of vessels or tumor. While an exact measurement and segmentation of critical structures extends beyond the scope of the current study, the establishment of the large change in volume provides adequate basis for this question to be evaluated in future experiments.

The present study was performed using a live porcine model, because it shows highest similarity to human anatomy. Still the porcine liver shows some noteworthy differences to the human one: it has four lobes instead of two and these can be flatter than in humans. Moreover, the porcine liver exhibits a different segmental nature [57]. Organ size was comparable to humans but less than in most western adult human livers [58]. The porcine model is well established in studies regarding the evaluation of the effects of pneumoperitoneum and for liver volumetry [7], whereas specific effects of respiration, pneumoperitoneum and laparotomy on liver volume had not been studied in detail in this model [25, 26, 33–35, 41, 59]. The application of the results in this study must be evaluated critically before using them in a clinical environment. The experimental nature of this study in a porcine model unfortunately results in a small study population (*n* = 10), and the resulting large confidence intervals must be acknowledged as a weakness. Nevertheless, the study showed a statistically significant change between all three measurements, as well as a statistically significant difference in liver volume between inspiratory and expiratory states. These results form the basis of the arguments put forth in this discussion, and other studies assessing preoperative planning and intraoperative volume show corroborating evidence [25, 51, 52, 58, 60–62]. Consistent with the recommendations to keep the number of animal experiments as low as possible, e.g., 3Rs (reduction, replacement, refinement) and PREPARE guidelines we chose to not perform additional experiments since the current number of animals was sufficient for statistical evaluation with the current focus of the study.

The gold standard for volumetry is water displacement of Archimedes, but as this is only possible with an explanted liver, we could not use this method. We used CT scans to assess liver volume as it is used as gold standard to preoperatively determine liver volume [60, 63, 64]. Image-based volumetric techniques especially regarding the measurement of liver volume are considered reliable and accurate [26, 58, 65–68]. Some studies however have shown that CT and MRI based methods seem to over- and underestimate in vivo liver volume from actual graft volume. These differences seem to be caused by measurements in different perfusion states and by inconsistencies in the method of image segmentation [61, 62, 69–74]. We used manual image segmentation of CT scans with semiautomated interpolation, as it provided accurate and consistent results and is used as the reference standard in many studies [26, 27, 57, 72, 75]. Nonetheless, intraobserver variability of up to 5% is reported, making the manual input a source of possible errors. The method of determining the inspiratory volumes was limited in that the respiration volume was defined by estimation and determined by visual control on the ventilation machine. A brief statement should be made here that the results of this study reflect respiratory states in mechanical ventilation, and not spontaneous respiration. However, for the purposes of the clinical applicability, this should not be seen as a large weakness of the study. The two main arguments of clinical relevance of this study stem from intraoperative assessment of resection volumes and from preoperative planning with CT images. In the case of intraoperative assessment, mechanical ventilation is an appropriate modality for the study. In preoperative CT scans, a breath hold will result in static intrathoracic pressure similar to the respiratory port occlusion used in the current study, however it must be noted that this is an approximation of the measurement, and not an exact replication of the clinical setting.

In conclusion, our results show that liver volume is influenced intraoperatively by pneumoperitoneum and laparotomy as well as by different inspiratory volumes. Changes of up to 13.2% in liver volume were found in the present experimental model between the pre- and intraoperative situation. Preoperative calculations and intraoperative navigation for major hepatic interventions should be done with consideration to these changes in volume. Furthermore, the results of this study can be used to help refine intraoperative navigation systems in hepatic surgery and interventions.

## Abbreviations

LDLT: Living donor liver transplantation
CT: Computed tomography
DICOM: Digital Imaging and Communications in Medicine
MITK: Medical Imaging Interaction Toolkit
